# Special Issue “Advances in Breast MRI”

**DOI:** 10.3390/diagnostics11122297

**Published:** 2021-12-08

**Authors:** Francesca Galati, Rubina Manuela Trimboli, Federica Pediconi

**Affiliations:** 1 Department of Radiological, Oncological and Pathological Sciences, Sapienza—University of Rome, 00161 Rome, Italy; francesca.galati@uniroma1.it; 2 Humanitas Clinical and Research Center, IRCCS, 20089 Rozzano, MI, Italy; rubina.trimboli@humanitas.it

**Keywords:** breast cancer, DWI, MR spectroscopy, MRI biomarkers, artificial intelligence

We thank all the authors, reviewers and the editorial staff who contributed to this Special Issue. The articles published in this Special Issue highlight how advances in breast magnetic resonance imaging (MRI) are improving breast cancer (BC) detection, characterization, prognosis and treatment. At present, BC is the most common female invasive cancer in the western countries and the commonest cause of cancer death in women [1]. About one in eight women will develop BC in their lifetime, with average age of onset declining over the years [2]. In spite of the increasing incidence, BC mortality progressively decreased in recent decades [3]. This reduction demonstrates the crucial relevance of early diagnosis in improving treatment strategies and outcomes [4]. To date, breast MRI represents the most sensitive technique for breast lesion detection. It is well-established and considered indispensable in breast imaging practice. Breast MRI consolidated indications are BC screening in women at increased risk, locoregional staging and neoadjuvant therapy monitoring [5]. In recent years, novel functional techniques, such as MR spectroscopy (MRS) and diffusion-weighted imaging (DWI), have been widely investigated to increase breast MRI accuracy and to provide deeper insights. DWI measures the water diffusivity of the tissues under examination and represents a valuable tool to distinguish benign from malignant breast lesions [6]. Moreover, considering the risks associated with the use of Gadolinium-based contrast agents, such as adverse reactions and brain deposition, DWI with ADC mapping has been also advocated as a stand-alone sequence for BC detection with interesting results [7,8,9]. However, bilateral DWI has limitations, such as magnetic susceptibility and chemical shift artifacts, low signal-to-noise ratio and low resolution [10]. Therefore, various approaches have been suggested for minimizing these drawbacks. Reduced field of view (rFOV) techniques obtain detailed images for a target region by reducing matrix size, leading to decreased susceptibility artifacts and increased spatial resolution at the expense of longer imaging time compared with single-shot EPI DWI. A few recent studies on breast imaging with rFOV DWI have shown that the images provide higher lesion conspicuity, better image quality and relatively higher resolution compared to images obtained using conventional bilateral DWI, and they can be potentially used instead of dynamic contrast-enhanced (DCE) MRI in BC patients [10,11,12,13]. Alternatively, advanced approaches based on DWI, such as intravoxel incoherent motion, diffusion weighted kurtosis and diffusion-tensor imaging, have recently emerged and are the current object of study, with promising results in terms of the improvement of BC diagnosis and subtype differentiation [6,14,15,16,17]. MRS is a noninvasive diagnostic tool able to assess metabolic information from a selected region within the tissue of interest based on the detection of the peak of some metabolites, such as the total choline peak (tCho) at 3.23 ppm. Elevated levels of tCho have been detected in malignant tumors, including BC, and are determined by the increased cell membrane turnover of neoplastic processes. Several studies have demonstrated that the inclusion of MRS in conventional breast MR examination improves diagnostic accuracy and reduces the number of unnecessary biopsies, since MRS has shown an adequate sensitivity and specificity in distinguishing between benign and malignant lesions [18,19,20,21]. Recent studies demonstrated that the detection of elevated tCho levels is associated with biological aggressive cancer phenotypes characterized by a high grade, large dimensions and high Ki67 proliferation rate [18,22]. In addition to tCho, in recent years MRS has been used to detect and monitor other metabolites, such as lipids, since lipid metabolism alterations were demonstrated to be associated with cancer development. Thakur et al. [23] confirmed the diagnostic and prognostic value of MRS, demonstrating that quantitative in vivo MRS assessment of lipid metabolism of breast lesions enables the identification of malignancies and the characterization of BC subtypes [24]. In this context precision medicine has developed, and the use of biomarkers to create customized treatments has progressively grown. In addition to traditional tissue sampling-derived biomarkers, nowadays imaging aims to offer a complementary method to obtain biological information about the hallmarks of cancer. Several perfusion parameters variably derived from breast MRI were initially correlated with traditional histological prognostic factors (grading, tumor size, HER2 expression and hormone receptors, Ki67 proliferation index), later with local recurrences, distant metastases and survival, opening a new scenario in the treatment of BC where MRI becomes an imaging biomarker. The ultimate goal is to noninvasively predict the phenotypic differences and molecular status of BC, which has become more and more essential for optimal treatment. In the last decade some authors demonstrated that different human cancer phenotypes of BC show specific imaging features. Triple-negative BCs are significantly associated with MRI intralesional necrosis and peritumoral edema on T2-weighted images [25,26,27,28], while rim enhancement in DCE MRI is an established finding of aggressiveness associated with increased angiogenesis, vascular endothelial growth factor expression and negative expression of estrogen and progesterone receptors [25,26]. On the other hand, irregular mass shape and not circumscribed margins are more frequently associated with luminal BCs, reflecting the lack of desmoplastic reaction and the relatively slow growth rate [29,30]. A growing interest is in the use of MRI as a prognostic tool helping to define prognosis as well as costumized therapy plans. The large datasets provided by and potentially extractable from breast MRI make it convenient for fitting artificial intelligence (AI) applications. When BC diagnosis or treatment planning are performed based on MRI data, radiologists are asked to integrate multiple information from multiple images. The landmark paper from Gilles et al. in 2016 shouted that “Images are more than pictures, they are data” and unmasked the hidden power of imaging methods encompassing information not always perceivable from human interpretation [31]. Machine learning methods can extract this information and analyze it with plenty of algorithms for a better understanding of the disease in vivo. Breast MRI represents a fertile ground for AI applications due its intrinsic multiparametric concept. Multiple image volumes for a single subject are generated, each containing proper data to be integrated and classified according to the specific diagnostic, therapeutic or prognostic aim. Breast MRI is playing an integral role both in clinical breast care and BC-related research. MRI is becoming essential for BC screening, diagnosis, staging and monitoring, and it offers one of the most appealing methods for the testing of AI and the manifestation of its potential. In the era of personalized medicine, with the fast-paced development of DWI and MRS, omics technologies, machine learning and big data, the role of imaging is being redefined to embrace new opportunities and to guide new approaches toward BC diagnosis and treatment [32,33].
